# Association between dietary insulin index and load with cardiometabolic risk factors and risk of metabolic syndrome among the patients with type 2 diabetes: a cross-sectional study

**DOI:** 10.1186/s40795-023-00803-z

**Published:** 2023-12-04

**Authors:** Vajeheh Arabshahi, Roksaneh Amiri, Samira Sadat Ghalishourani, Nazila Hasaniani, Shadi Nozarian, Ronia Tavasolian, Alireza Khiabani, Mehran Rahimlou

**Affiliations:** 1 Department of Nutrition, School of Public health, Iran University of Medical Sciences, Tehran, Iran; 2 Department of Nutrition, School of Public Health, Zanjan University of Medical Sciences, Azadi Square, Zanjan, Iran; 3 Department of Physical education and sport science, science of research branch, Islamic Azad University, Tehran, Iran; 4 Emam Reza Hospital affiliated with Social Security Organization, Urmia, Iran; 5 Department of Nutrition, Ahvaz Jondishapur University of Medical Sciences, Ahvaz, Iran; 6 School of Medicine, Bam University of Medical Sciences, Bam, Iran; 7 Department of Nutrition, School of Medicine, Urmia University of Medical Sciences, Urmia, Iran

**Keywords:** Dietary insulin index, Dietary insulin load, T2DM, Metabolic syndrome, Hyperglycemia

## Abstract

**Background:**

This study aims to investigate the association between dietary insulin index (DII) and load (DIL) with cardiometabolic risk factors and the risk of developing metabolic syndrome (MetS) among patients with type 2 diabetes (T2DM).

**Methods:**

A cross-sectional study was conducted among 500 T2DM patients. Dietary intake was assessed using a validated food frequency questionnaire, and DII and DIL were calculated based on insulin response and energy content. Logistic regression analyses were performed to determine the odds ratios (ORs) for MetS.

**Results:**

Participants in the highest quartile of DIL had significantly higher odds of MetS (OR: 2.16; 95% CI: 1.02–4.25, P = 0.039) and hyperglycemia (OR: 1.69; 95% CI: 1.08–4.96, P = 0.032). We also discovered that patients in the highest quartile of DII had higher odds of MetS (OR: 1.69; 95% CI: 1.08–4.96, P = 0.034) and hyperglycemia (OR: 1.39; 95% CI: 1.04–4.12, P = 0.019). Furthermore, participants in the highest quartile of DIL (OR: 1.64; 95% CI: 1.00-2.59, P = 0.03) and DII (OR: 1.42; 95% CI: 1.05–1.95, P = 0.026) had higher odds of high waist circumference. When it came to hypertriglyceridemia, we found a significant association between DII and DIL only in the crude model, not the fully adjusted model. However, we didn’t observe any significant association between DII and DIL with hypercholesteremia, Low HDL, and high blood pressure (P > 0.05).

**Conclusion:**

Our study provides evidence suggesting that a higher DII and DIL may be associated with an increased risk of cardiometabolic risk factors and MetS in patients with T2DM.

## Introduction

T2DM is a prevalent chronic condition characterized by impaired insulin function and elevated blood glucose levels [1]. The worldwide incidence of T2DM is increasing significantly, with approximately 415 million adults diagnosed in 2015 [2]. By 2045, it is projected that this number will rise to an estimated 629 million people worldwide [3]. This trend poses significant challenges to individuals’ overall health and well-being. Managing T2DM requires a multifaceted approach, incorporating lifestyle modifications and dietary interventions. With the global increase in T2DM prevalence, exploring the relationship between dietary factors and cardiometabolic risk factors has become paramount [4].

Although numerous metabolic and lifestyle factors are recognized contributors to the onset of T2DM and its associated complications, there is a growing interest in the role of environmental factors in the development of this disease [5]. Increasingly, a substantial body of evidence indicates that insulin resistance plays a significant role in the pathogenesis of T2DM [6]. Insulin resistance, a condition where insulin secretion is diminished or insulin is unable to efficiently facilitate the transportation of glucose into the body’s peripheral tissues, is crucial in the development of both MetS and T2DM [7, 8]. Several studies have shown a relationship between postprandial insulin changes and the occurrence of various chronic diseases. Elevated blood sugar levels after eating and the resulting excessive insulin levels are believed to play a role in the onset and progression of conditions such as obesity, diabetes, cardiovascular disease, and MetS [9, 10].

The potential of food to trigger the release of insulin after a meal is crucial when considering the prevention and control of insulin resistance and T2DM. While the glycemic index (GI) of foods can provide valuable insight into how they affect blood sugar levels based on their carbohydrate content, it cannot accurately predict the corresponding insulin response for the majority of food items [11]. One such dietary factor of interest is the DII and DIL. The DII measures the insulinemic potential of various foods based on their macronutrient composition, allowing for a more nuanced understanding of their impact on postprandial glucose and insulin response [12]. Similarly, DIL focuses on the overall insulin response elicited by the entire diet. The concept of DII refers to a measurement that directly assesses the post-meal insulin response to a specific food, comparing it to an equal energy portion of a reference food (similar to how the glycemic index uses either glucose or white bread as a comparison) [13, 14]. The use of the DII is considered more appropriate for examining the connection between chronic disease development and food intake compared to the GI because the DII specifically measures the insulin response. By utilizing the DII, we can establish the DIL by multiplying the DII of each food by its energy content and the frequency of consumption [15].

Various studies showed the relationship between DII and DIL with chronic diseases such as certain cancers [16], obesity [17], diabetes [18] and other diseases, with some results being contradictory.

Understanding the relationship between DII and load and their association with cardiometabolic risk factors in T2DM is crucial for optimizing management and preventing complications. Identifying dietary patterns that promote better glycemic control and mitigate cardiovascular risk factors can significantly impact the quality of life for individuals living with T2DM. Therefore, this study is designed to investigate the association between DII and load with cardiometabolic risk factors among patients with T2DM. Additionally, we evaluated the risk of developing MetS, a cluster of cardiometabolic abnormalities associated with increased cardiovascular morbidity and mortality.

## Materials and methods

### Participants and study design

This cross-sectional study was conducted among patients with T2DM referred to the specialized diabetes clinic of Zanjan University of Medical Sciences, Iran. Patients were included in the study if they met the following criteria: (1) diagnosed with T2DM, (2) adults aged between 18 and 70 years, and either receiving oral anti-diabetic medication or not receiving any pharmacological treatment. The diagnosis of T2DM in this study adhered to the criteria set by the American Diabetes Association, which classifies a person as having T2DM if they meet either of the following conditions: a fasting plasma glucose level equal to or higher than 126 mg/dl or an oral glucose tolerance test result equal to or higher than 200 mg/dl [19]. The study excluded pregnant and lactating women, patients using insulin, individuals under special diets within the past year, those with abnormal calorie intake (less than 800 kcal or more than 4200 kcal), individuals with autoimmune diseases such as celiac disease or type 1 diabetes, those who experienced hyperglycemia-induced ketoacidosis within the last month, people with cancer, cognitive diseases like Alzheimer’s or Parkinson’s, individuals with grade 2 obesity (BMI ≥ 35), non-cooperative participants who did not complete the questionnaire, and patients who had used antioxidant and anti-inflammatory supplements in the past three months. The sample size was calculated according to the study by Abaj et al [20]. Based on this, we determined the required sample size by considering a type 1 error of 5% (α = 0.05), the standard deviation of the dietary insulin index equal to 5.38 in diabetic patients, and a 95% confidence interval. According to this calculation, we needed 445 individuals for the present cross-sectional study. However, the current analysis involved data from 500 participants.

### Ethics approval

The study was conducted in accordance with the Declaration of Helsinki, and the research protocol received approval from the Ethics Committee of Zanjan University of Medical Sciences (Ethics Code: IR.ZUMS.REC.1402.009). Before collecting data, participants were provided with a detailed explanation of the study’s objectives and methods, and informed consent was obtained from all subjects.

### Dietary intake assessment

We assessed participants’ dietary intakes using a reliable block-format 120-item food frequency questionnaire (FFQ) validated for adults in Iran [21, 22]. Specifically designed for Iranian adults, the questionnaire’s reliability and validity have been confirmed by previous studies investigating the links between diet and disease [17, 23, 24]. Each participant had the FFQ completed by an experienced interviewer. Participants provided dietary intake information, specifying whether it was for a day, week, month, or year, and indicated their intake based on the serving size of each food item. To enhance estimate precision, interviewers displayed household measures or serving sizes of each food item to participants. We determined the daily intake in grams for each food item by considering its consumption frequency and serving size. Additionally, we computed the daily nutrient intake for each participant by considering the nutrient content of all consumed foods, relying on the national nutrient databank of the US Department of Agriculture [25].

### Dietary insulin index and load

The food insulin index represents the increase in insulin response measured over a 2-hour period when consuming a 1,000-kJ (239 kcal) portion of the test food, relative to the insulin response generated by a 1,000-kJ (239 kcal) portion of the reference food. The insulin index for 68 different food items was gathered from prior studies conducted by Bao et al. (50 items) [26], Bell et al. (13 items) [27], and Holt et al. (5 items) [28]. The insulin index of three food items, namely tea, coffee, and salt, was determined to be zero due to their extremely low levels of energy, carbohydrates, protein, and fat. The insulin index measures how much a particular food item increases insulin levels after consumption. In this case, these three items have a negligible impact on insulin secretion. To account for the 49 food items not included in the referenced studies’ food lists, the insulin index of comparable food items was estimated using their energy, fiber, carbohydrate, protein, and fat content correlation. This approach allowed for an approximation of the insulin index values for those foods based on their nutritional composition similarities. Take dates and raisins as an example, both being dried fruits with comparable energy, carbohydrate, fat, protein, and fiber content. Consequently, we chose to use the insulin index of raisins to estimate the index for dates, given the similarities in nutritional composition between the two fruits. To determine the DIL, the initial step involved calculating the insulin load of each food. This was done by multiplying the insulin index of the food by the energy content per 1 gram of the food and further multiplying it by the amount of that food consumed in grams per day. The DIL for each individual was determined by adding up the insulin load of all the consumed foods. Subsequently, the DII for each participant was computed by dividing their DIL by their total energy intake [29].

### Biochemical assessment

For biochemical evaluation, 10 cc of fasting blood was collected from all patients. All experiments were conducted in the central laboratory of Zanjan University of Medical Sciences by a skilled technician. Serum concentrations of blood sugar, total cholesterol, and triglycerides were measured using enzymatic methods with standard kits (Pars Azmoon, Tehran, Iran). HDL-C concentrations were determined by measuring the precipitation of lipoproteins containing apo B using phosphotungstic acid. LDL concentration was calculated using Friedewald’s formula: LDL-c (mg/dL) = TC (mg/dL) − HDL-c (mg/dL) − TG (mg/dL)/5 [30].

To measure the SBP and DBP, patients were initially asked to sit and rest for 10 min. Each patient’s systolic and diastolic blood pressure was then measured twice by an expert, and the average of the two readings was recorded as the systolic or diastolic blood pressure. Blood pressure was measured using a mercury manometer (Micro life AG, 9443 Widnau / Switzerland).

### Anthropometric and physical activity evaluation

A questionnaire, assessing physical activity using the Metabolic Equivalent of Task (MET) system, was employed to gauge participants’ engagement in physical activities [31]. Participants’ height and weight were measured using Seca equipment from Germany, with a precision of 0.1 kg for weight and 0.5 cm for height. Measurements were taken while participants were dressed in lightweight clothing, without shoes. Waist circumference was determined with a flexible tape at the midpoint between the lowest rib and iliac crest, with an accuracy of 0.1 cm. Body mass index (BMI) was calculated by dividing weight in kilograms by the square of the height in meters.

### Statistical analysis

The patients were categorized into groups based on the quartile thresholds of their DIL and DII scores. To examine variations in quantitative variables across the quartiles of DIL and DII, one-way analysis of variance was used. Additionally, for categorical data, a Chi-Square test was utilized. Binary logistic regression with adjusted models was employed to calculate the odds ratio (OR) and 95% confidence interval (CI) for MetS, considering the quartiles of DIL and DII scores. MetS was determined according to the criteria outlined in the modified Iranian National Cholesterol Education Program for Adults [32, 33], where the presence of MetS was indicated by having at least three of the following components: (1) abdominal obesity (waist circumference ≥ 95 cm for both sexes), (2) low HDL-C levels (< 50 mg/dL [1.293 mmol/L] for women and < 40 mg/dL [1.034 mmol/L] for men), (3) high TG levels (> 150 mg/dL [1.694 mmol/L]), (4) abnormal glucose regulation (FBG ≥ 100 mg/dL [5.550 mmol/L]), and (5) elevated blood pressure (systolic blood pressure > 130 mm Hg and diastolic blood pressure ≥ 85 mm Hg).

Different models of binary logistic regression were used: Model 1, adjusted for age, sex, and energy intake, and Model 2, adjusted for sex, energy intake, BMI, physical activity, and duration of diabetes. In all models, participants in the first quartiles of DIL and DII were considered as the reference group. The categories of DIL and DII were treated as ordinal variables in the binary logistic regression to capture the trend in odds ratios across different quartiles. Statistical analyses were performed using SPSS version 18 (SPSS Inc, Chicago, IL). A significance level of P < 0.05 was used to determine statistical significance.

## Results

### General characteristics of participants

Table 1 displays the overall traits of participants across different quartiles of DII and DIL. In this study, information from a total of 500 patients with T2DM was examined, with a mean age of 46.93 ± 10.85 years and a mean BMI of 28.76 ± 2.66 kg/m2. Two hundred and twenty-eight participants (45.6%) were male, and the rest were female. Additionally, 70% of the participants were married, and 22.6% were smokers. The average duration of diabetes among the participants was 5.13 ± 3.37 years. In terms of physical activity levels, 53.8% of patients were less active, 34% were moderately active, and 12.2% were active. No significant differences were observed between patients across the DII and DIL quartiles in terms of the mentioned variables (P > 0.05).

**Table 1:** The general status of the variables examined in the study in the quartiles of insulin index and insulin load

	Quartiles of DIL					Quartiles of DII				
Variables	Q1 (125)	Q2 (125)	Q3 (125)	Q4 (125)	***P***-value^d^	Q1 (125)	Q2 (125)	Q3 (125)	Q4 (125)	***P***-value^d^
Quartiles range	< 150,354	150,355–184,670	184,671–228,350	> 228,350		< 46.33	46.34–67.98	67.99–93.49	> 93.50	
Age (years)	46.59 ± 11.03	47.05 ± 10.65	46.95 ± 11.98	47.25 ± 11.33	0.59	46.21 ± 11.31	47.23 ± 10.88	46.10 ± 10.9	47.21 ± 11.13	0.63
Sex (male)	57 (45.6%)	57(45.6%)	58(46.4%)	56(44.8%)	0.74	58 (46.4%)	55(44%)	60 (48%)	55 (44%)	0.42
BMI (kg/m^2^)	28.51 ± 2.13	28.29 ± 2.40	29.21 ± 3.12	28.73 ± 2.56	0.63	28.26 ± 2.56	28.15 ± 2.33	29.10 ± 3.06	28.12 ± 2.85	0.51
Weight (kg)	79.96 ± 11.31	79.12 ± 12.10	81.08 ± 12.46	80.41 ± 11.67	0.61	80.58 ± 12.41	78.38 ± 11.95	81.12 ± 12.65	80.40 ± 11.93	0.49
Waist circumference (cm)	92.4 ± 9.43	93.81 ± 10.23	94.12 ± 11.38	94.83 ± 10.75	0.37	92.65 ± 9.51	93.76 ± 10.43	94.5 ± 10.98	94.92 ± 11.25	0.29
Marital Status (Married)	87(69.6%)	90(72%)	84(67.2%)	89(71.2%)	0.73	89(71.2%)	84(66.4%)	92(73.6%)	85(68%)	0.71
Duration of diabetes (years)	5.17 ± 3.93	4.92 ± 2.17	5.72 ± 3.11	5.04 ± 3.58	0.47	5.38 ± 3.19	4.49 ± 2.79	5.95 ± 3.65	4.88 ± 2.83	0.55
Smoking (yes)	28(22.4%)	30(24%)	25(20%)	30(24%)	0.43	32(25.6%)	25(20%)	27(21.6%)	25(20%)	0.38
Family history of diabetes (%)	64.37	60.21	72.35	69.47	0.28	62.7%	59.8%	73.4%	68.9%	0.16
Physical Activity (number)										
Less active people	69	67	68	65	0.44	68	65	70	68	0.35
Moderate active	40	45	41	44		40	46	39	42	
Active	16	13	16	16		17	14	16	15	
Calorie intake (kcal/day)	2525.65± 309.11	2761.30± 369.70	2500± 355.92	2670.43± 369.40	0.55	2542.45± 283.56	2612.19± 345.89	2419.12± 340.51	2543.25± 283.56	0.42
Carbohydrate (gram/day)	300.29± 129.95	290.78± 123.75	339.45± 139.55	342.55± 141.65	0.32	302.5± 132.43	282.67± 128.44	320.29± 144.55	340.51± 143.56	0.26
Fat (gram/day)	73.62± 28.48	78.44± 32.26	83.34± 32.63	81.5± 30.96	0.48	75± 29.45	76.65± 30.63	82.23± 38.44	80.93± 30.41	0.46
Protein (gram/day)	87.30± 27.69	89.72± 23.40	85.22± 26.11	84.32± 25.43	0.69	85.47± 21.33	88.90± 25.49	87.37± 24.53	83.40± 23.62	0.71

^a^ DIL: dietary insulin load; ^b^DII: Dietary insulin index; ^c^ data are presented as standard deviation or percent; ^d^, Obtained from analysis of variance or Chi-squared test, where appropriate

Regarding dietary intake, the average calorie intake was 2630 ± 347.66 kcal/day. Additionally, no significant differences were found among the DIL and DII quartiles in terms of carbohydrate, protein, and fat intake (P > 0.05).

### Biochemical variables differences in quartiles of dietary insulin index and insulin load

The mean concentration of biochemical indices across quartiles of DII and DIL is summarized in Table 2. Participants in the highest quartile of DIL had significantly higher FBS levels compared to those in the lowest quartile (158.74 ± 30.13 mg/dl vs. 130.12 ± 19.38; P = 0.04). Additionally, higher levels of total cholesterol (P = 0.021) and DBP (P = 0.04) were found among patients in the highest quartile of DIL than in the lowest quartile. However, no significant differences were observed in terms of TG (P = 0.33), LDL (P = 0.29), HDL (P = 0.34), and SBP (P = 0.12).

**Table 2:** Biochemical variables differences in quartiles of dietary insulin index and insulin load

	Quartiles of DIL					Quartiles of DII				
Variables	Q1 (125)	Q2 (125)	Q3 (125)	Q4 (125)	***P***-value^d^	Q1 (125)	Q2 (125)	Q3 (125)	Q4 (125)	***P***-value^d^
FBS (mg/dl)	130.12± 19.38	133.15± 23.45	146.55± 26.18	158.74± 30.13	**0.04**	133.32± 18.45	137.61± 22.51	144.76± 19.67	159.77± 24.67	**0.036**
TG (mg/dl)	158.33± 19.15	155.19± 20.63	158.23± 24.39	164.39± 19.22	0.33	160.65± 27.32	159.32± 21.96	155.28± 25.38	165.19± 30.12	0.27
Cho (mg/dl)	191.97± 25.44	215.18± 23.76	236.19± 24.47	256.26± 26.37	**0.021**	197.17± 29.33	216.32± 33.19	229.32± 34.36	248.36± 32.93	**0.024**
LDL (mg/dl)	130.25± 18.73	128.33± 20.19	132.41± 24.29	136.27± 23.21	0.29	132.19± 20.14	126.53± 18.76	135.17± 23.47	138.35± 22.73	0.18
HDL (mg/dl)	42.63± 9.26	43.96± 8.21	42.21± 10.15	42.63± 9.26	0.34	42.29± 7.84	45.09± 8.56	43.06± 8.83	42.85± 10.97	0.2
SBP	124.26± 16.96	119.41± 14.37	124.64± 18.68	123.67± 14.79	0.12	122.08± 15.40	124.72± 19.10	121.19± 13.21	123.95± 17.16	0.46
DBP	81.79± 11.83	78.82± 10.53	79.04± 11,33	83.25± 10.47	**0.04**	80.06± 11.02	83.53± 12.81	80.81± 11.29	82.14± 11.51	0.33

^a^ DIL: dietary insulin load; ^b^DII: Dietary insulin index; ^c^ data are presented as standard deviation or percent; ^d^, Obtained from analysis of variance or Chi-squared test, where appropriate, FBS: fasting blood sugar; Cho: total cholesterol; LDL: low density lipoprotein; HDL: high density lipoprotein; SBP: systolic blood pressure; DBP: diastolic blood pressure

Regarding DII, our analysis results showed that diabetic patients in the highest quartile of DII had significantly higher concentrations of FBS (P = 0.036) and cholesterol (P = 0.024) than those in the lowest quartile. However, no significant differences were found across the quartile of DII in terms of TG (P = 0.27), LDL (P = 0.18), HDL (P = 0.2), SBP (P = 0.46), and DBP (P = 0.33).

### Correlation of DII and DIL with odds of cardiometabolic risk factors

The results of the current study indicated that there was no significant correlation between DIL and the likelihood of MetS in the initial analysis (Table 3) (OR: 1.81; 95% CI: 0.43–1.95, P = 0.07). However, after adjusting for certain factors in Model 1 (OR: 2.59; 95% CI: 1.05–6.48, P = 0.04) and Model 2 (OR: 2.16; 95% CI: 1.02–4.25, P = 0.039), it was reported that patients in the highest quartile of DIL had significantly higher odds of experiencing MetS compared to those in the lowest quartile.

**Table 3:** Multivariate-adjusted odds ratio (95% CI) for MetS, hyperglycemia, hyperlipidemia and hypertension across quartiles of DIL^a^ and DII^b^ scores

	Quartiles of DIL					Quartiles of DII				
Variables^c^	Q1 (125)	Q2 (125)	Q3 (125)	Q4 (125)	***P***-value^d^	Q1 (125)	Q2 (125)	Q3 (125)	Q4 (125)	***P***-value^d^
**MetS** ^q^										
Crude	Ref	1.75(0.86–3.27)	1.06(0.55–1.94)	1.81(0.43–1.95)	0.07	Ref	1.77(0.94–3.31)	1.70(0.43–2.30)	1.63(0.83- 3.12)	0.08
Model 1	Ref	1.22(0.41–3.54)	1.73(0.64–4.43)	2.59(1.05–6.48)	**0.04**	Ref	1.25(0.36–2.85)	1.97(0.32–2.65)	1.72(1.15–4.22)	**0.02**
Model 2	Ref	1.47(0.83–3.94)	1.63(0.58–4.39)	2.16(1,02-4.25)	**0.039**	Ref	1.52(0.69–3.11)	1.86(0.72–2.09)	2.03(1.08–4.73)	**0.034**
**Hyperglycemia** **(FBS > 100 mg/dl)**										
Crude	Ref	0.67(0.07–2.71)	0.88(0.18–4.17)	1.64(1.04–4.48)	**0.048**	Ref	1.13(0.24–5.35)	0.92(0.14–5.81)	1.55(1.12–4.53)	**0.03**
Model 1	Ref	0.71(0.29–1.56)	1.04(0.2–5.04)	1.83(1.10–5.12)	**0.039**	Ref	1.04(0.19–4.69)	0.88(0.2–6.13)	1.46(1.06–3.72)	**0.027**
Model 2	Ref	2.10(0.91–4.85)	1.15(0.48–2.75)	1.69(1.08–4.96)	**0.032**	Ref	1.07(0.15–7.28)	1.27(0.12–8.43)	1.39 (1.04–4.12)	**0.019**
**Hypertriglyceridemia** **(TG > 150 mg/dl)**										
Crude	Ref	0.96(0.36–2.87)	1.13(0.41–2.08)	2.19(1.12–4.63)	**0.034**	Ref	2(0.75- 5.29)	1.41(0.54–3.68)	1.21(1.15–5.78)	**0.02**
Model 1	Ref	0.85(0.36–2.28)	1.06(0.43–2.63)	1.76(1.09–3.82)	**0.025**	Ref	1.90 (0.67–5.39)	1.33(0.48–3.87)	1.25(1.17–5.02)	**0.017**
Model 2	Ref	0.93(0.33–2.82)	1.02(0.36–2.89)	1.75(0.98–3.76)	0.08	Ref	1.10(0.26–4.72)	1.81(0.28–3.15)	2.56(0.84–5.63)	0.12
**Hypercholesteremia** **(Cho > 200 mg/dl)**										
Crude	Ref	0.6(0.28–1.35)	0.68(0.29–1.60)	0.58(0.26–1.37)	0.6	Ref	1.27(0.56–2.86)	0.81(0.33–1.97)	0.92(0.39–2.19)	0.76
Model 1	Ref	0.67(0.29–1.56)	0.71(0.38–1.73)	0.62(0.32–1.69)	0.74	Ref	1.21(0.53–2.92)	0.86(0.45–2.12)	0.88(0.38–2.31)	0.56
Model 2	Ref	0.64(0.23–1.49)	0.69(0.37–1.57)	0.65 (0.27–1.73)	0.62	Ref	1.30 (0.49–3.41)	0.73(0.23–2.40)	0.81(0.22–2.60)	0.85
**Low HDL** **(HDL < 40 mg/dl)**										
Crude	Ref	1.73(0.81–3.70)	1.01(0.45–2.23)	1.26(0.52–3.03)	0.46	Ref	1.23(0.57–2.62)	0.81(0.33–1.93)	1.27(0.55–2.89)	0.72
Model 1	Ref	1.80 (0.58–4.15)	1.05(0.38–2.38)	1.28 (0.48–3.19)	0.43	Ref	1.28 (0.55–2.84)	0.76(0.3–1.88)	1.23(0.48–2.76)	0.65
Model 2	Ref	2.10(0.91–4.85)	1.15(0.48–2.75)	1.33(0.52–3.36)	0.31	Ref	1.76(0.7–4.36)	1.16(0.37–3.64)	2.06 (0.63–6.23)	0.41
**High Blood** **Pressure (> 130/85)**										
Crude	Ref	1.02(0.39–2.68)	2.73(0.93–6.69)	1.52(0.63–3.68)	0.19	Ref	1.18(0.44–5.19)	1.13(0.4–5.19)	1.47(0.86–6.10)	0.33
Model 1	Ref	1.31(0.45–3.78)	2.13(0.81–5.62)	1.61(1.13–6.56)	**0.026**	Ref	1.13 (0.32–5.84)	1.01 (0.29–5.32)	1.88(0.45–5.84)	0.39
Model 2	Ref	1.22(0.4–3.65)	3.59(0.1–5.21)	1.15(0.75–6.19)	0.14	Ref	1.53 (0.12–5.39)	1.56(0.13–5.37)	1.52(0.12–5.39)	0.42
**High WC** **(WC > 95 cm)**										
Crude	Ref	0.87(0.69–1.08)	0.99(0.81–1.23)	1.07(0.89–1.32)	0.65	Ref	0.93(0.76–1.17)	1.24(0.96–1.49)	1.29(1.04–1.60)	**0.01**
Model 1	Ref	1.02(0.78–1.33)	1.20(0.86–1.79)	1.35(0.85–1.99)	0.27	Ref	0.92(0.69–1.12)	1.25(0.98–1.50)	1.28(1.01–1.84)	**0.02**
Model 2	Ref	1.20(0.9–1.57)	1.53(1.05–2.08)	1.64(1.00-2.59)	**0.03**	Ref	1.05(0.79–1.33)	1.39(1.08–1.81)	1.42(1.05–1.95)	**0.026**

^a^ DIL, dietary insulin load; ^b^DII, Dietary insulin index; ^c^ Data are odds ratio and 95% confidence interval; ^d^, obtained from binary logistic regression, FBS, fasting blood sugar; Cho, total cholesterol; LDL, low density lipoprotein; HDL, high density lipoprotein; SBP, systolic blood pressure; DBP, diastolic blood pressure;WC, waist circumference; q, clarified based on the Iranian modified National Cholesterol Education Program for Adults. Model 1, adjusted for age, sex and energy intake and Model 2, adjusted for sex and energy intake, BMI, physical activity, and duration of diabetes

Additionally, both the initial and fully adjusted models showed that participants in the highest quartile of DIL exhibited significantly higher odds of hyperglycemia (OR for fully adjusted model: 1.69; 95% CI: 1.08–4.96, P = 0.032). Furthermore, a significant correlation between DIL and the likelihood of hypertriglyceridemia was found only in the initial and Model 1 adjusted analysis (OR for Model 1 adjustment: 1.76; 95% CI: 1.09–3.82, P = 0.025), but this relationship lost its significance in the second adjusted model. In terms of high blood pressure, a significant relationship was only observed in Model 1 (OR: 1.61; 95% CI: 1.13–6.56, P = 0.026). However, no significant relationship was found between DIL quartiles and hypercholesteremia or low HDL levels (P > 0.05). Finally, in relation to WC, patients in the highest DIL quartile showed a significant likelihood of having a high WC compared to those in the lowest quartile, but this was only observed in the fully adjusted model (OR: 1.64; 95% CI: 1.00-2.59, P = 0.03).

On the other hand, patients in the highest DII quartile had a significantly higher likelihood of having MetS in both Model 1 and the fully adjusted model (OR for the fully adjusted model: 1.69; 95% CI: 1.08–4.96, P = 0.034). Additionally, it was revealed that patients in the highest DII quartile had notably higher odds of experiencing hyperglycemia in both the crude and adjusted models (OR: 1.39; 95% CI: 1.04–4.12, P = 0.019). In the Model 1 adjustment, participants in the highest DII quartile had a 25% greater risk of hypertriglyceridemia compared to those in the lowest quartile of DII (OR: 1.25; 95% CI: 1.17–5.02, P = 0.017). However, after accounting for potential confounding factors in the fully adjusted model, the association disappeared. Regarding high WC, patients in the highest DII quartile had a 42% higher risk of having a high WC than individuals in the lowest quartile (OR: 1.42; 95% CI: 1.05–1.95, P = 0.026).

## Discussion

The present study explored the association between DII and DIL with cardiometabolic risk factors and the risk of developing MetS among patients with T2DM. The findings suggest a significant relationship between higher DII and DIL and an increased risk of cardiometabolic risk factors and MetS in individuals with T2DM.

Recently, there has been notable interest in the DII, which has proven to be a more effective predictor of risks related to chronic conditions when compared to the GI. Importantly, DII depends directly on insulin response rather than other mediators [27, 34]. In this current study, a positive correlation was noted between DII and DIL scores and the risk of MetS. Consistent with our results, a population-based cohort study conducted in Iran reported that men in the third quartile of DIL had 61% greater odds for having MetS compared with those in the first quartile. After adjusting for potential confounding factors, women in the highest quartile of DIL exhibited a 77% increased likelihood of having MetS compared to women in the lowest quartile and similar findings were observed for DII [29]. Another investigation involving 203 overweight/obese adolescents found that those in the upper tertile of DIL faced a significantly higher risk of metabolically unhealthy obesity compared to individuals in the lower tertile [35]. MetS stands out as a prevalent global health issue [36] interlinked with other chronic conditions like cardiovascular diseases [37] and certain cancers [38]. Abdominal obesity and insulin resistance stand out as the primary risk factors for MetS. In a cross-sectional study, Mirmiran et al. demonstrated a notable positive correlation between higher scores of DII and DIL and the risk of insulin resistance [15]. In another prospective study lasting three years and involving 927 men and women, a positive correlation was observed between DIL and an elevated likelihood of insulin resistance [18].

While the comprehensive understanding of the precise mechanisms by which dietary insulin indices influence the development of cardiometabolic risk factors remains elusive, there have been reports indicating that the consumption of a diet with a high insulinemic potential may enhance insulin secretion, thereby resulting in heightened carbohydrate oxidation and diminished fat oxidation. As a result, this type of diet may facilitate the accumulation of fat, especially in the abdominal region, increasing the risk of abdominal obesity, insulin resistance and an unfavorable metabolic profile [39]. Additionally, given that potentially high insulinemic foods are swiftly digested, absorbed, and converted into glucose, they would result in a rapid elevation in blood glucose and blood insulin levels. As a result, this would lead to a reduction in glucose excursion. The abrupt drop in blood glucose levels would cause a decrease in the feeling of fullness, bringing back the sensation of hunger [18]. Consequently, this can result in an increase in calorie intake, potentially raising the risk of developing abdominal obesity and an unfavorable metabolic profile. Additionally, elevated DIL and DII scores are correlated with insulin resistance and decreased C-peptide levels, respectively, suggesting a possible connection to β-cell dysfunction [40, 41].

In this study, a notable association was identified between DII and DIL and the risk of hyperglycemia and elevated WC. As reported in an observational study by Joslowski et al., heightened DII and DIL during puberty were linked to elevated levels of body fat percentage in young adulthood [42]. Prolonged elevated levels of DIL and DII are proposed to lead to diminished insulin sensitivity, decreased lipolysis, and the encouragement of body fat accumulation. Moreover, heightened DIL and DII may potentially enhance body fat by triggering the secretion of both insulin and insulin growth factor-1 (IGF-1), promoting the growth of preadipocytes and ultimately resulting in the formation of body fat [42].

In the current study, it has been reported a positive linear correlation between DII and DIL with hypertriglyceridemia in the crude model and after controlling for age, sex and energy intake. In line with these findings, both the Nurses’ Health Study and the Health Professionals Follow-up Study indicated that adhering to a diet characterized by high DII and DIL was associated with increased serum levels of TG [43]. The mechanisms behind these findings are probably multifaceted. One potential explanation is that diets with higher DII and DIL may trigger more pronounced postprandial glucose and insulin responses, resulting in prolonged hyperglycemia and dyslipidemia [35, 43].

The present study had several strengths. The study involves a substantial sample size of 500 patients with T2DM, enhancing the statistical power and reliability of the findings. Also, the study employs adjusted models, including factors such as age, sex, energy intake, BMI, physical activity, and duration of diabetes. This enhances the robustness of the results by controlling for potential confounding variables, allowing for a more accurate assessment of the association between DII, DIL, and cardiometabolic risk factors. However, it is important to note that our study has certain limitations. The cross-sectional design of the study limits its ability to establish causation. While associations between DII, DIL, and cardiometabolic risk factors are identified, the temporal sequence of events cannot be determined. Also, the reliance on self-reported dietary intake through a food frequency questionnaire may introduce recall bias and may not fully capture the nuances of participants’ eating habits. Additionally, self-reporting might lead to underestimation or overestimation of actual food consumption. Furthermore, we did not account for potential confounding factors, such as medication use and socioeconomic status, which could have an impact on the identified associations.

## Conclusion

In conclusion, our study provided evidence supporting the association between elevated DII and DIL with an increased risk of cardiometabolic risk factors and Metabolic Syndrome (MetS) among patients with T2DM. Specifically, participants in the highest quartiles of both DII and DIL demonstrated higher odds of MetS, hyperglycemia, and increased waist circumference. These findings highlighted the potential role of dietary insulin indices in influencing metabolic health outcomes in individuals with T2DM. Alao, our results suggested that diets with a higher insulinemic potential, as indicated by elevated DII and DIL scores, may contribute to unfavorable metabolic profiles, including insulin resistance and abdominal obesity. Future investigations could explore the following areas: First, conducting longitudinal studies to establish causal relationships and better understand the temporal sequence of events between dietary insulin indices and the development of cardiometabolic risk factors. Second, implementing dietary interventions to assess the impact of modifying dietary insulin indices on metabolic outcomes in individuals with T2DM and finally, conducting mechanistic studies to elucidate the biological pathways through which dietary insulin indices influence insulin resistance, abdominal obesity, and other cardiometabolic risk factors.

## Data Availability

The data and all supporting materials used in the preparation of this manuscript are freely available from the corresponding author at reasonable request.

## References

[1] Galicia-Garcia U (2020). Pathophysiology of type 2 Diabetes Mellitus. Int J Mol Sci.

[2] Cho NH et al. *IDF Diabetes Atlas: Global estimates of diabetes prevalence for 2017 and projections for 2045* Diabetes research and clinical practice. 2018;138:271–281. 10.1016/j.diabres.2018.02.023 29496507

[3] Tinajero MG, Malik VS (2021). An update on the epidemiology of type 2 Diabetes: a global perspective. Endocrinol Metabolism Clin.

[4] Williams DM, Jones H, Stephens JW (2022). Personalized type 2 Diabetes management: an update on recent advances and recommendations.

[5] Taylor R (2013). Type 2 Diabetes: etiology and reversibility. Diabetes Care.

[6] Olokoba AB, Obateru OA, Olokoba LB (2012). Type 2 Diabetes Mellitus: a review of current trends. Oman Med J.

[7] Schofield CJ, Sutherland C (2012). Disordered insulin secretion in the development of insulin resistance and type 2 Diabetes. Diabet Med.

[8] Savage DB, Petersen KF, Shulman GI (2007). Disordered lipid metabolism and the pathogenesis of insulin resistance. Physiol Rev.

[9] O’Keefe JH, Bell DS (2007). Postprandial hyperglycemia/hyperlipidemia (postprandial dysmetabolism) is a cardiovascular risk factor. Am J Cardiol.

[10] Standl E, Schnell O, Ceriello A (2011). Postprandial hyperglycemia and glycemic variability: should we care?. Diabetes Care.

[11] Vlachos D (2020). Glycemic index (GI) or glycemic load (GL) and dietary interventions for optimizing postprandial hyperglycemia in patients with T2 Diabetes: a review. Nutrients.

[12] Yari Z (2020). New insight into Diabetes management: from glycemic index to dietary insulin index. Curr Diabetes Rev.

[13] Hajhashemy Z (2022). Dietary insulin index and insulin load in relation to hypertriglyceridemic waist phenotype and low brain derived neurotrophic factor in adults. Front Nutr.

[14] Vajdi M (2023). Dietary insulin index and load and cardiometabolic risk factors among people with obesity: a cross-sectional study. BMC Endocr Disorders.

[15] Mirmiran P (2015). Dietary insulin load and insulin index are associated with the risk of insulin resistance: a prospective approach in tehran lipid and glucose study. J Diabetes Metab Disord.

[16] Farvid MS (2021). Postdiagnostic dietary glycemic index, glycemic load, dietary insulin index, and insulin load and Breast cancer survival. Cancer Epidemiol Biomarkers Prev.

[17] Anjom-Shoae J (2020). Association between dietary insulin index and load with obesity in adults. Eur J Nutr.

[18] Anjom-Shoae J (2023). Dietary insulin index and load in relation to cardiometabolic risk factors in patients with type 2 Diabetes Mellitus: a cross-sectional study on the RaNCD cohort study. Nutrition.

[19] Association AD (2020). 2. Classification and diagnosis of Diabetes: standards of medical care in diabetes—2020. Diabetes Care.

[20] Abaj F, Rafiee M, Koohdani F (2021). Interaction between CETP polymorphism and dietary insulin index and load in relation to cardiovascular risk factors in diabetic adults. Sci Rep.

[21] Esfahani FH (2010). Reproducibility and relative validity of food group intake in a food frequency questionnaire developed for the Tehran lipid and glucose study. J Epidemiol.

[22] Mirmiran P (2010). Reliability and relative validity of an FFQ for nutrients in the Tehran lipid and glucose study. Public Health Nutr.

[23] Ghorabi S (2019). Association between the DASH diet and metabolic syndrome components in Iranian adults. Diabetes Metab Syndr.

[24] Sharif Y (2021). Legume and nuts consumption in relation to odds of Breast Cancer: a case-control study. Nutr Cancer.

[25] USDA N. *United States department of agriculture* Natural Resources Conservation Service. Plants Database. http://plants.usda.gov (accessed in 2000), 1999.

[26] Bao J (2011). Prediction of postprandial glycemia and insulinemia in lean, young, healthy adults: glycemic load compared with carbohydrate content alone. Am J Clin Nutr.

[27] Bell KJ (2016). Algorithms to improve the prediction of postprandial insulinaemia in response to common foods. Nutrients.

[28] Holt S, Miller J, Petocz P (1997). An insulin index of foods: the insulin demand generated by 1000-kJ portions of common foods. Am J Clin Nutr.

[29] Sadeghi O (2020). Dietary insulin index and dietary insulin load in relation to metabolic syndrome: the Shahedieh cohort study. J Acad Nutr Dietetics.

[30] Knopfholz J et al. *Validation of the friedewald formula in patients with metabolic syndrome* Cholesterol. 2014;261878. 10.1155/2014/261878 PMC3941209 24672715

[31] Ainsworth BE (2000). Compendium of physical activities: an update of activity codes and MET intensities. Med Sci Sports Exerc.

[32] AZIZI F et al. *Appropriate definition of metabolic syndrome among Iranian adults: report of the Iranian National Committee of Obesity* 2010. 20804311

[33] Grundy SM et al. *Clinical management of metabolic syndrome: report of the American Heart Association/National Heart, Lung, and Blood Institute/American Diabetes Association conference on scientific issues related to management* Circulation. 2004;109(4):551–556. 10.1161/01.CIR.0000112379.88385.67 14757684

[34] Sung K-C (2018). Obesity and incidence of Diabetes: Effect of absence of metabolic syndrome, insulin resistance, inflammation and fatty liver. Atherosclerosis.

[35] Hajhashemy Z (2022). Association of dietary insulin index and dietary insulin load with metabolic health status in Iranian overweight and obese adolescents. Front Nutr.

[36] Bovolini A (2021). Metabolic syndrome pathophysiology and predisposing factors. Int J Sports Med.

[37] Silveira Rossi JL (2022). Metabolic syndrome and Cardiovascular Diseases: going beyond traditional risk factors. Diab/Metab Res Rev.

[38] Battelli MG (2019). Metabolic syndrome and cancer risk: the role of xanthine oxidoreductase. Redox Biol.

[39] Kahn SE, Hull RL, Utzschneider KM (2006). Mechanisms linking obesity to insulin resistance and type 2 Diabetes. Nature.

[40] Hellström PM (2013). Satiety signals and obesity. Curr Opin Gastroenterol.

[41] Llewellyn CH (2014). Satiety mechanisms in genetic risk of obesity. JAMA Pediatr.

[42] Joslowski G (2012). Prospective associations of dietary insulin demand, glycemic index, and glycemic load during puberty with body composition in young adulthood. Int J Obes.

[43] Nimptsch K (2011). Dietary insulin index and insulin load in relation to biomarkers of glycemic control, plasma lipids, and inflammation markers. Am J Clin Nutr.

